# Risk factors associated with age at onset of Parkinson’s disease in the UK Biobank

**DOI:** 10.1038/s41531-023-00623-9

**Published:** 2024-01-02

**Authors:** Yuanfeng Huang, Qian Chen, Zheng Wang, Yijing Wang, Aojie Lian, Qiao Zhou, Guihu Zhao, Kun Xia, Beisha Tang, Bin Li, Jinchen Li

**Affiliations:** 1 National Clinical Research Centre for Geriatric Disorders, Department of Geriatrics, Xiangya Hospital, Central South University, Changsha 410008, Hunan China; 2 Bioinformatics Center, Xiangya Hospital & Furong Laboratory, Central South University, Changsha 410008, Hunan China; 3 Department of Oncology, Xiangya Hospital, Central South University, Changsha 410008, Hunan China; 4 Department of Neurology, Xiangya Hospital, Central South University, Changsha 410008, Hunan China; 5 National Health Commission Key Laboratory of Birth Defect Research and Prevention, Hunan Provincial Maternal and Child Health Care Hospital, Changsha 410008, Hunan China; 6 Centre for Medical Genetics & Hunan Key Laboratory of Medical Genetics, School of Life Sciences, Central South University, Changsha 410008, Hunan China

**Keywords:** Parkinson's disease, Risk factors

## Abstract

Substantial evidence shown that the age at onset (AAO) of Parkinson’s disease (PD) is a major determinant of clinical heterogeneity. However, the mechanisms underlying heterogeneity in the AAO remain unclear. To investigate the risk factors with the AAO of PD, a total of 3156 patients with PD from the UK Biobank were included in this study. We evaluated the effects of polygenic risk scores (PRS), nongenetic risk factors, and their interaction on the AAO using Mann–Whitney *U* tests and regression analyses. We further identified the genes interacting with nongenetic risk factors for the AAO using genome-wide environment interaction studies. We newly found physical activity (*P* < 0.0001) was positively associated with AAO and excessive daytime sleepiness (*P* < 0.0001) was negatively associated with AAO, and reproduced the positive associations of smoking and non-steroidal anti-inflammatory drug intake and the negative association of family history with AAO. In the dose-dependent analyses, smoking duration (*P* = 1.95 × 10^−6^), coffee consumption (*P* = 0.0150), and tea consumption (*P* = 0.0008) were positively associated with AAO. Individuals with higher PRS had younger AAO (*P* = 3.91 × 10^−5^). In addition, we observed a significant interaction between the PRS and smoking for AAO (*P* = 0.0316). Specifically, several genes, including *ANGPT1* (*P* = 7.17 × 10^−7^) and *PLEKHA6* (*P* = 4.87 × 10^−6^), may influence the positive relationship between smoking and AAO. Our data suggests that genetic and nongenetic risk factors are associated with the AAO of PD and that there is an interaction between the two.

## Introduction

Parkinson’s disease (PD) is a heterogeneous disorder caused by genetic, environmental, and aging factors^1–3^. Substantial evidence has shown that age at onset (AAO) is a major cause of clinical heterogeneity in patients with PD^4,5^. The incidence of late-onset PD (LOPD) is five- to tenfold higher than that of early-onset PD (EOPD) and is similar between males and females^6^. Compared to LOPD, EOPD is characterized by slower progression, a decreased risk of developing dementia, reduced concentrations of biomarkers, a lower risk of non-motor symptoms, and an increased rate of dyskinesia in response to levodopa^7–9^. The pathological underpinnings of EOPD and LOPD also differ^10,11^. Therefore, the development of PD is closely associated with the AAO. However, the mechanisms underlying AAO heterogeneity remain largely unknown.

Substantial evidence has revealed a close high correlation between genetic factors and AAO. For example, PD patients with monogenetically inherited variants often present with an early onset^12,13^. Previous large genome-wide association studies (GWAS) and meta-analyses have identified several significant variants associated with AAO. Among them, rs356203 (*SNCA*), rs983361 (*SNCA*), rs34311866 (*TMEM175*), rs4698412 (*BST1*), and rs9356013 (*PARK2*) could reduce the AAO, whereas rs9783733 (*NDN*) could delay the AAO^14–17^. Moreover, the polygenic risk score (PRS) constructed using cumulative small-effect variants from PD GWAS is thought to be negatively correlated with the AAO^18,19^. Importantly, the heritability of the AAO is only approximately 0.11, which is much lower than the risk of PD^14^, suggesting that other factors may contribute to the AAO.

In addition to genetic factors, epidemiological studies have shown that nongenetic risk factors influence AAO^20^. For instance, AAO has been observed to be delayed in patients with PD who smoke, consume coffee, and in those prescribed aspirin^21^. Furthermore, two published studies have demonstrated the potential influence of nongenetic risk factors on AAO in PD patients with the *LRRK2* p.G2019S or *GBA* p.N370S mutation^22,23^. However, previous studies on the AAO merely focused on the interaction effect of a special single nucleotide polymorphism (SNP) with nongenetic risk factors, usually without considering the interaction effect between them at a genome-wide level.

In this study, we used data from a large-scale UK Biobank cohort to identify new risk factors for AAO in patients with PD. We further performed regression analyses to test the interaction effects of the PD PRS and nongenetic risk factors. Additionally, the possible gene interacting with nongenetic risk factors for AAO heterogeneity was identified using genome-wide environment interaction studies (GWEIS).

## Results

### Demographics of study samples

A total of 3786 individuals with PD were included in this study. After excluding non-Europeans and related individuals, 3156 of 3768 PD cases remained, of which 3063 PD cases had a Hospital Episode Statistics-coded diagnosis. As shown in Supplementary Table 1, 62.57% of the PD patients were male, the median age at recruitment (AAR) was 64 (interquartile range [IQR] = 60–67) years, the median AAO was 70 (IQR = 63–75) years, and the median Townsend Deprivation index was −2.3007 (IQR = −3.7274–0.4774).

### Nongenetic risk factors associated with the AAO of PD

Based on simple group comparisons, patients with PD who reported smoking, moderate or vigorous physical activity (> = 600 metabolic-equivalent task (MET)-min/week), and non-steroidal anti-inflammatory drugs (NSAIDs) intake had a later AAO (Table 1). However, patients with PD with excessive daytime sleepiness (EDS) or a family history of PD had a younger AAO. After adjusting for confounders, generalized linear regression models showed similar results. The exclusion of self-reported PD cases did not alter the results (Supplementary Table 2).

**Table 1:** Association of nongenetic risk factors with AAO of PD.

	Yes	No	*P*	Adjusted_*P*
Normal BMI				
*N*	872	2257		
Median AAO (IQR)	70.0 (62.0–75.0)	70.0 (63.0–75.0)	0.0574	0.6744
Smoking				
*N*	1439	1702		
Median AAO (IQR)	71.0 (65.0–76.0)	69.0 (61.0–75.0)	<0.0001*	0.0001*
Coffee consumption				
*N*	2533	617		
Median AAO (IQR)	70.0 (63.0–75.0)	70.0 (62.0–74.0)	0.0899	0.9830
Tea consumption				
*N*	2725	421		
Median AAO (IQR)	70.0 (63.0–75.0)	70.0 (63.0–75.0)	0.9658	0.2038
Alcohol consumption				
*N*	2990	159		
Median AAO (IQR)	70.0 (63.0–75.0)	70.0 (63.0–76.0)	0.9843	0.3720
Cheese consumption				
*N*	2963	97		
Median AAO (IQR)	70.0 (63.0–75.0)	71.0 (66.0–75.0)	0.2742	0.1240
Milk consumption				
*N*	3056	97		
Median AAO (IQR)	70.0 (63.0–75.0)	71.0 (64.0–75.0)	0.7554	0.2740
Moderate or vigorous physical activity				
*N*	1742	771		
Median AAO (IQR)	71.0 (64.0–75.0)	69.0 (62.0–74.0)	<0.0001*	<0.0001*
NSAIDs intake				
*N*	1014	2097		
Median AAO (IQR)	72.0 (65.0–76.0)	70.0 (62.0–74.0)	<0.0001*	0.0093*
EDS				
*N*	1159	1980		
Median AAO (IQR)	69.0 (61.0–75.0)	71.0 (64.0–75.0)	<0.0001*	<0.0001*
Family history of PD				
*N*	280	2876		
Median AAO (IQR)	68.0 (60.0–74.0)	71.0 (63.0–75.0)	0.0001*	0.0001*

*Median AAO* median age at onset of PD, *IQR* interquartile range, N number of individuals, *Normal BMI:* *yes* person with normal BMI, *Normal BMI: no* person with abnormal BMI, *Smoking: yes* person currently or ever smoke, *Smoking:* *no* person never smoke, *Coffee consumption: yes* person drink more than zero cup of coffee each day, *Cofee consumption: no* person drink zero cup of coffee each day, *Tea consumption: yes* person drink more than zero cup of tea each day, *Tea consumption: no* person drink zero cup of tea each day, *Alcohol consumption: yes* person currently or ever drink, *Alcohol consumption: no* person never drink, *Cheese consumption: yes* person eat cheese, *Cheese consumption: no* person do not eat cheese, *Milk consumption: yes* person drink milk, *Milk consumption: no* person do not drink milk, *Moderate or vigorous physical activity: yes* person with metabolic-equivalent task (MET) minutes per week more than or equal to 600, *Moderate or vigorous physical activity: no* person with metabolic-equivalent task (MET) minutes per week less than 600, *NSAIDs intake: yes* person regularly take in aspirin or ibuprofen, *NSAIDs intake: no* person do not take in any aspirin or ibuprofen, *P* two-sided exploratory *P* values from Mann–Whitney *U* tests, *Adjusted_P* two-sided exploratory *P* values from generalized linear models adjusted for AAR, gender, and Townsend Deprivation index.

Significant associations (*P* < 0.05) are in bold with star.

In the investigation of the dosage effects of smoking, alcohol consumption, coffee consumption, tea consumption and physical activity on AAO, nonparametric Spearman’s correlation analysis showed that smoking duration (*r* = 0.1138, *P* = 0.0027), daily cups of tea consumption (*r* = 0.0675, *P* = 0.0004) and the intensity of physical activity (*r* = 0.0623, *P* = 0.0021) were positively associated with the AAO (Table 2). After adjusting for AAR, sex, and Townsend Deprivation index, generalized linear regression models showed smoking duration (*β* = 0.0789, *P* = 1.95 × 10^−6^), daily cups of coffee consumption (*β* = 0.1623, *P* = 0.0150), and daily cups of tea consumption (*β* = 0.1669, *P* = 0.0008) were significantly associated with older AAO.

**Table 2:** Dose-dependent analyses of nongenetic risk factors on AAO of PD.

Variables	Spearman’s correlation analyses		Generalized linear regression models		
	*r*	*P*	*β*	SE	Adjusted_*P*
Smoking duration	0.1138	0.0027*	0.0798	0.0166	**1.95** **×** **10**^−6^*
Alcohol consumption	−0.0006	0.9928	−0.0024	0.0234	0.9190
Coffee consumption	0.0067	0.7369	0.1623	0.0667	0.0150*
Tea consumption	0.0675	0.0004*	0.1669	0.0497	0.0008*
Physical activity	0.0623	0.0021*	8.82 × 10^−5^	5.56 × 10^−5^	0.1130

*r* Spearman’s rank correlation coefficient, *P* Spearman’s exploratory *P* value, *β* regression coefficient from generalized linear models, *SE* standard error, *Adjusted_P* two-sided exploratory *P* values from generalized linear models adjusted for AAR, gender, and Townsend Deprivation index.

Significant associations (*P* < 0.05) are in bold with stars.

### Higher polygenic risk scores are associated with earlier AAO of PD

**Fig. 1:**
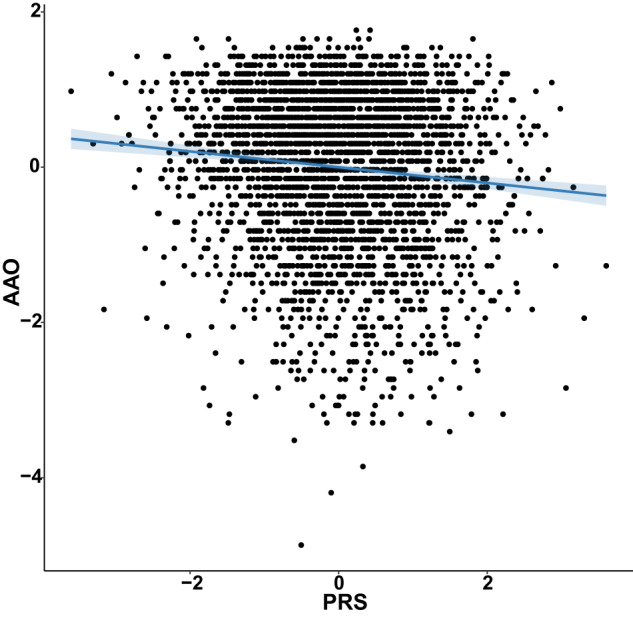
Association of AAO and PRS in PD. Z-standardized AAO and PRS for each study participant with the generalized linear regression model. The one point represents one participant. The blue line represents generalized linear regression analysis was used to assess the association between them. The blue shade represents the 95% confidence interval of the regression coefficients of generalized linear model.

The generalized linear regression model adjusted for AAR, sex, Townsend Deprivation index, and first four principal components (PCs1-4) revealed an inverse association between AAO and PRS. As shown in Fig. 1, for one standard deviation (SD) increase in the PRS, the AAO was approximately advanced about 0.5 years (*β* = −0.0567, SE = 0.0138, *P* = 3.91 × 10^−5^).

### The effect of smoking on AAO of PD depends on genetic risk stratification

We selected nongenetic risk factors with adjusted P values less than 0.05 (smoking, moderate or vigorous physical activity, NSAIDs intake, EDS, and family history of PD) for analyzing the interaction effect with PRS (Table 3). There was a significant multiplicative interaction between PRS and smoking (*β* = 0.5300, *P* = 0.0316). There was also weaker evidence for a multiplicative interaction between a family history of PD (*P* = 0.0524) and PRS, as well as EDS (*P* = 0.0599). There was no interaction effect between the PRS and nongenetic risk factors in the other models. These results suggested that smoking was a potential protective factor against AAO among individuals with a higher genetic risk.

**Table 3:** Interaction effects of PRS and nongenetic risk factors on AAO of PD.

Model number	Variable	Estimate	SE	*t* value	*P*
1	Smoking	0.9681	0.2524	3.8350	0.0001
	PRS	−0.7556	0.1671	−4.5210	<0.0001
	Smoking × PRS	0.5348	0.2487	2.1500	0.0316*
2	Moderate or vigorous physical activate	1.3308	0.2953	4.5060	<0.0001
	PRS	−0.0906	0.2373	−0.3820	0.7027
	Moderate or vigorous physical activate × PRS	−0.5422	0.2897	−1.8710	0.0614
3	NSAID=s intake	0.6942	0.2687	2.5840	0.0098
	PRS	−0.5125	0.1516	−3.3800	0.0007
	NSAIDs intake × PRS	0.0045	0.2680	0.0170	0.9866
4	EDS	−2.4980	0.2542	−9.8260	<0.0001
	PRS	−0.3187	0.1580	−2.0170	0.0438
	EDS × PRS	−0.4719	0.2507	−1.8820	0.0599
5	Family history of PD	−1.7108	0.4356	−3.9280	<0.0001
	PRS	−0.5699	0.1295	−4.4010	<0.0001
	Family history of PD × PRS	0.8614	0.4439	1.9410	0.0524

*P* two-sided exploratory *P* values from generalized linear models adjusted for AAR, gender, Townsend Deprivation index, and PCs1-4.

Significant associations (*P* < 0.05) are in bold with stars.

**Fig. 2:**
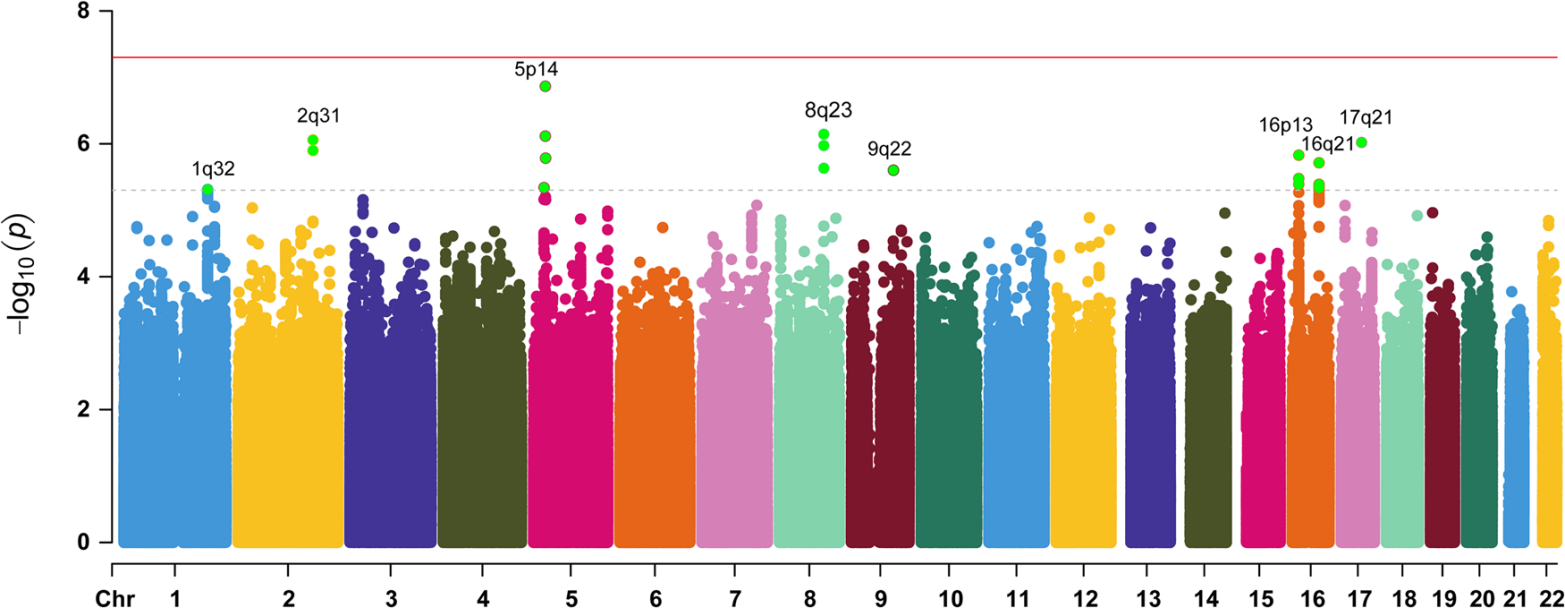
Manhattan plot for GWEIS analysis for AAO of PD. The *X* axis shows the chromosomal location, the *Y* axis shows the negative logarithm of the association *P* value for each SNP. The red line denotes the *P* value threshold for genome-wide significance (*P* = 5 × 10^−8^), while the gray line denotes the *P* value threshold for suggestive significance (*P* = 5 × 10^−6^).

Given the significant interaction between smoking and PRS in the regression analysis, GWEIS analysis was performed to investigate the potential interaction effect of SNPs and smoking on the AAO. A quantile-quantile plot showed genomic inflation factor λ was 0.972, indicating that population stratification had a negligible effect on the overall significance of the effect estimates (Supplementary Fig. 1). We detected eight loci, harboring several candidate genes, such as *ANGPT1* (*P* = 7.17 × 10^−7^) and *PLEKHA6* (*P* = 4.87 × 10^−6^), which showed suggestive significant interactions with smoking in the AAO (Supplementary Table 3). A visualization of the results is shown in Fig. 2.

### Functional annotations of the loci interacting with smoking

When mining the gene expression database, we found that some of the eight suggestive significant loci found in this GWEIS were highly significantly expressed quantitative trait loci for genes in the substantia nigra, cortex, and basal ganglia (Supplementary Table 4). For example, the lead SNP (rs10093967) at 8q23 and its proxy were intronic variants in *ANGPT1* and cis-eQTLs for *ANGPT1* and *OXR1* in the occipital and frontal cortices. We further searched for co-localization with the regulatory elements of the lead SNPs and their proxies. Remarkably, these lead sites and their proxies have been reported to be associated with PD or smoking, including *DBX2*, *MEF2*, and *EGR-1*, among others^24–26^. The lead *PLEKHA6* SNP and its proxy were mapped to transcription factor-binding sites, promoter histone marks in the skeletal muscle, enhancer histone marks in the brain substantia nigra, and DNase I hypersensitivity sites in the fetal brain. Finally, an investigation of the Phenoscanner database^27^ revealed that lead SNPs and proxies were also associated with DNA methylation levels in the peripheral blood and cord blood. As shown in Supplementary Table 5, the C allele of rs4951056 at 1q32 and rs9934772 at 16q21 were associated with higher levels of methylation in cord blood, while the C allele of rs10093967 at 8q23 was associated with lower level of methylation in cord blood. In addition, the C allele of rs4951056 at 1q32 and rs10093967 at 8q23 were also associated with higher level of methylation in human immune cells, while the C allele of rs9934772 at 16q21 and the A allele of rs113991597 were associated with lower levels of methylation in human immune cells.

## Discussion

We analyzed the independent and combined effects of genetic and nongenetic risk factors on the AAO of PD in a large-scale European cohort. As a result, we not only found significant new associations between physical activity and EDS with AAO, but also replicated the significant effects of smoking, NSAIDs intake, family history of PD, and PRS. We also observed significant dose-dependent associations of smoking duration, coffee consumption, and tea consumption with older AAO. We further demonstrated that the effect of smoking on the AAO depends on the genetic risk stratification. Notably, we identified several candidate genes that could influence the positive association between smoking and AAO at the genome-wide level.

Many epidemiological studies have highlighted the protective role of smoking in PD. But due to limited sample size, it is unclear whether smoking delays the AAO^21,28^. A similar controversy has been observed in patients with a family history of PD^29–31^. This study further provided some evidence to support that smoking is related to the later AAO of PD, while family history of PD is related to the earlier AAO of PD in European patients with PD.

Nicotine in cigarettes can exert protective effects against damage to nigral dopaminergic neurons by: (1) stimulating the release of dopamine in different regions of the brain to increase dopamine levels^32,33^; (2) suppressing dopamine transporter activity^34^; (3) reducing neuroinflammatory and cellular stresses via inhibition of SIRT6 expression^35^; (4) preventing glutamate-induced neurotoxicity^36^; (5) suppressing 1-methyl-4-phenyl-1,2,3,6-tetrahydropyridine (MPTP) induced dopaminergic neuron degeneration^37^; (6) suppressing free-radical damage and monoamine oxidase B^38^; and (7) suppressing the unfolded protein response^39^. However, the influence of other cigarette components remains unclear. Based on the clinical diagnostic criteria developed by the Movement Disorder Society^40^, the age at which PD patients first develop cardinal motor features is recorded as the AAO. However, only a loss of more than 50% of dopaminergic neurons results in the symptoms and manifestations of PD^41^. Therefore, the neuroprotective effects of nicotine may delay the onset of PD symptoms and signs.

Interestingly, we found that coffee and tea consumption showed positive associations with older AAO only in dose-dependent analyses but not in dichotomized exposure analyses. This may be caused by the small sample size of non-consumers.

Various studies support the neuroprotective effects of coffee and tea on PD through caffeine^42,43^. As an antagonist of the adenosine A2a receptor, caffeine could enhance dopamine transmission^44^. Caffeine also could modulate neuroinflammation, excitotoxicity, and mitochondrial function^45^. There were also other bioactive components of tea presenting neuroprotective effects, including tea polyphenols, theanine and theaflavins^46^. Interestingly, some studies have suggested the protective effect of tea is specific to black tea^47^. A two-sample Mendelian randomization study also did not verify the association between green tea intake and AAO^48^.

Although NSAIDs intake have been found to delay the AAO in the United States population^21^, our study is the first to demonstrate their protective effect in the European population. Notably, significant new effects of physical activity and EDS on the AAO have been reported for the first time.

Physical activity can modify synaptic connectivity within the dopamine-depleted striatum^49^, directly increasing stimulus-evoked dopamine release or decreasing dopamine decay, which improves behavioral outcomes in rodent neurotoxicant-induced models^50,51^. In addition, physical activity has been shown to suppress oxidative stress and promote the expression of neurotrophic factors^52^, thereby potentially attenuating dopaminergic neuron degeneration and contributing to the neuroplasticity and survival of dopaminergic neurons^53,54^. In contrast, EDS is thought to be a potential prodromal symptom of PD with similar neuropathological lesions. On the one hand, according to Braak’s theory^55^, EDS is associated with neuronal loss in areas closely involved in sleep and wake coordination, such as the hypothalamus, coeruleus, and ascending reticular activating system^55,56^, occurring in stage II of PD before substantia nigra degeneration. In contrast, noradrenergic, serotonergic, and dopaminergic deficits may contribute to the development of EDS^57^.

Next, we explored the association between PRS and AAO, consistent with previous findings^14,15^, we found that an increase of PRS was associated with an earlier AAO in patients of PD. The finding suggested that the known genetic composition of PD can partially explain the heterogeneity of AAO.

Furthermore, we assessed the role of nongenetic risk factors that may contribute to the AAO in different strata of genetic risk according to the PRS. We observed that smoking had a greater protective effect against the AAO in those with a higher genetic risk. As previous studies have reported, some genetic factors could interact with smoking in PD or with its AAO^58,59^. An involvement of nicotine dependent gene, *CHRNA5*, was reported to modify the association between smoking and AAO^60^. Another study showed that smoking was only associated with a later AAO in PD patients with *LRRK2* p.G2019S carriers^23^. These studies suggested that there could be real differences in the protective effect of smoking on the AAO between different stratum of genetic risk.

We identified eight loci that harbored relevant candidate genes that interacted with smoking in the AAO at the genome-wide level, with biological functions associated with PD or exposure to smoking. The lead SNP at 1q32 is located in an intron of *PLEKHA6*, playing an important role in regulating copper homeostasis, whereby disturbed copper homeostasis interferes with dopamine metabolism, oxidative stress, and α-synuclein aggregation leading to the occurrence of PD^61–63^. An epigenome-wide association study confirmed that this gene co-localizes with smoking-related differentially methylated sites^64,65^. The lead SNP at 8q23, located in an intron of *ANGPT1*, significantly decreased the expression level after exposure to cigarette smoke extract, but increased its expression in GBA-related PD^66,67^. The lead SNP at the 2q31 locus is located near *ITGA4*, which shows upregulated gene expression after exposure to two known components of cigarettes, arsenic and BaP^68^. Moreover, lead SNPs and their proxies were observed to co-localize with several transcription factor-binding sites that showed biologically relevant functions in PD. The dysregulation of *MEF2* function may underlie PD pathogenesis and DNA methylation changes related to tobacco^26,69^. *EGR-1* has been shown to promote neuroinflammation and dopaminergic cell body loss in the substantia nigra pars compacta of the MPTP-PD mouse model^70^. It has been confirmed that the expression of *EGR-1* responded to cigarette smoke in pulmonary epithelial cells^24^. Therefore, candidate genes at these loci are not only strongly related to PD but also to smoking, which confirms the reliability of our results. Furthermore, we observed differentially methylated CpG sites near the lead SNPs, some of which were associated with smoking. However, the relationship between genetic variants, gene expression, and methylation is complex and further research is required.

The strength of this study is that we used a large cohort to investigate the relationship between genetic factors, nongenetic risk factors, and their interactions with the AAO of PD in the European population. As many nongenetic risk factors are influenced by sex, age, and other factors, we adjusted for relevant confounding factors as much as possible. To avoid sample overlap, we used the standard PRS calculated by Thompson et al. using five GWAS datasets and excluding the UK Biobank samples^71^. Simultaneously, a proportion of PD cases were self-reported, which may have led to an inaccurately reported AAO and the possibility of recall bias. Another important consideration is that a younger AAO correlates with a younger AAR. Although the results did not change after excluding self-reported cases in the sensitivity analysis and adjusting for AAR as a confounder, errors caused by recall bias could not be eliminated. Finally, whether the results of this study can be applied to other ethnic groups needs to be carefully considered, as we only included Europeans.

In conclusion, in addition to several known nongenetic risk factors, this study found new effects of physical activity and EDS on the AAO in patients with PD. At the same time, we found that the protective effect of smoking depended on the genetic risk, and several candidate genes, such as *ANGPT1* and *PLEKHA6*, may play an important role in disease onset. This study provides some evidence for a combined association between genetic and nongenetic risk factors and the AAO of PD.

## Methods

### Study population

This research has been conducted using the UK Biobank Resource under approved application numbers 103082. The UK Biobank recruited above 500,000 UK residents from 22 assessment centers between 2006 and 2010. Detailed genetic, health, environmental, and lifestyle information was collected from the cohort. Details of the UK Biobank have been described previously^72^. The UK Biobank has ethics approval from the North West Multi-Centre Research Ethics Committee and all UKB participants provided written informed consent. PD patients were defined using the International Statistical Classification of Diseases and Related Health Problems 10th Revision (ICD10) code G20 from data field 41202, and self-reported PD cases with code 1262 were identified from data field 20002. The AAO for PD was derived from the date of the first G20 report. One individual was randomly selected from a pair of related individuals with first-, second-, or third-degree relatives. In addition, individuals of non-European ancestry were excluded to reduce bias due to demographic histories.

### Risk factors

We included several risk factors reported to be associated with PD in the UK Biobank. Candidate variables included body mass index (BMI), smoking status, alcohol consumption, coffee consumption, tea consumption, cheese consumption, milk consumption, physical activity, NSAIDs intake, EDS, and family history of PD. All those factors were obtained through a self-completed touchscreen questionnaire or physical measurements at baseline; specific details are as follows:

BMI was calculated from the height and weight measured during the visit to the initial assessment center. Based on the World Health Organization’s criteria^73^, BMI was divided into two categories: normal (18.5–24.9 kg/m^2^) and abnormal (<18.5 kg/m^2^ or >=25 kg/m^2^).

The participants were asked about their smoking status (never, previous, or current) and were defined as non-smokers (never smokers) or as smokers. For smokers, the duration of smoking was estimated according to the years from the start of smoking until smoking cessation, or from the age at starting smoking until the diagnosis of PD. At the same time, we took the duration of smoking as a continuous variable to analyze the association between the duration of smoking and AAO in smokers.

At baseline, participants reported their alcohol consumption status (never, previously, or currently). Participants were defined as non-drinkers if they had never consumed alcohol. The remaining participants were defined as drinkers. In addition, participants reported their weekly alcohol consumption. According to the Health Survey for England methods protocol^74^, we converted alcohol consumption per week to units of pure alcohol per week^75^. At the same time, we took the units of pure alcohol per week as a continuous variable to analyze the association between the dose of pure alcohol per week and AAO in drinkers.

The participants reported their daily number of cups of coffee consumed, including less than one or a specific number per day. Coffee consumers were defined as those who drank more than zero cup of coffee daily. At the same time, we took the total cups of daily coffee consumption as a continuous variable to analyze the association between the dose of coffee consumption and AAO in coffee consumers.

The participants reported their daily number of cups of tea consumed, including less than one or a specific number per day. Tea consumers were defined as those who drank more than zero cup daily. At the same time, we took the total cups of daily tea consumption as a continuous variable to analyze the association between the dose of tea consumption and AAO in tea consumers.

The participants answered questions regarding the details of their medication use. NSAIDs intake was defined as a regular intake of aspirin or ibuprofen. Regular use was defined as NSAIDs on most days of the week more than the last 4 weeks.

The participants were asked about their frequency of cheese consumption. Cheese non-consumers were defined as those who never ate cheese, whereas the others were defined as cheese consumers.

Based on the type of milk the participants drank, they were regarded as non-milk consumers if they reported never or rarely consuming milk.

The participants’ total physical activity was assessed using the International Physical Activity Questionnaire. Participants were divided into three groups based on the MET-min/week: low physical activity (<600 MET-min/week), moderate physical activity (600–3000 MET-min/week), and high physical activity (≥ 3000 MET-min/week)^76^. The threshold at 600 MET-min/week was regarded as the recommended guideline for moderate physical activity. At the same time, we took the total physical activity as a continuous variable to analyze the association between the dose of physical activity and AAO after excluding participants with a total physical activity of zero.

The EDS of the participants was assessed based on the likelihood of unintentionally dozing off or falling asleep during the day. Participants were not considered to have EDS if they reported that they never or rarely dozed off or fell asleep during the daytime.

Participants were asked if their first-degree relatives had PD. First-degree relatives include fathers, mothers, and siblings. Individuals were defined as having a family history of PD if any of their first-degree relative were recorded to have PD.

### Polygenic risk score

Thompson et al. used data from five GWAS, excluding UK Biobank samples, to construct a standard PRS for individuals, and published the results in the UK Biobank PRS Release for all UK Biobank researchers to access^71^. In addition, genetic PCs were also calculated by the UK Biobank.

### Statistical analysis

We excluded participants who chose the option “do not know” or “prefer not to answer,” when filling out the questionnaire, as well as those who had missing data. The Mann–Whitney *U* tests were used to assess the different distributions of AAO among the different subgroups. Nonparametric Spearman’s correlation analyses were performed to assess whether there was a dosage effect of the variables on the AAO. Generalized linear regression models were built to evaluate independent and combined associations between nongenetic risk factors and PRS. In the analysis of the correlation between the PRS and AAO, the AAO was converted into Z-scores to make the analysis results more easily interpretable. We used inverse normal transformation to transform the PRS and regarded the PRS as a continuous variable in the interaction analysis. All results were adjusted for AAR, sex, and Townsend Deprivation index. For the PRS and interaction analysis, PCs1–4 were also included as covariates. All statistical analyses and diagrams were performed using R version 4.1.3.

### Genome-wide environment interaction study

To explore the interaction effect of SNPs and smoking on AAO, we used a generalized linear regression model in PLINK2.0 (V.2.00aLM 64-bit Intel)^77^. AAR, sex, and the PCs1–4 of the population structure were used as covariates. We excluded variants that deviated from the Hardy–Weinberg equilibrium (*P* < 1e-6), variants with a call rate <95%, rare variants with a minor allele frequency < 1%, and low imputation quality (*R*^2^ < 0.6). Individuals with more than 5% missing genotypes were excluded from the study. After filtering, 9,231,448 variants and 3124 PD cases remained in the GWEIS. The significance threshold was set at *P* = 5.0 × 10^−8^. *P* = 5.0 × 10^−6^ was set as a suggestive significance threshold.

### Functional annotations of the loci interacting with nongenetic risk factors

We evaluated the biological characteristics of these loci detected by GWEIS. For each locus, we integrated the most significant variant and its proxies with a strong linkage imbalance (*r*^2^ >= 0.8). To explore the potential influence of the new loci identified in this study on expression traits, we searched for cis-expression quantitative trait loci (eQTLs) in several gene expression databases containing different organizations, including brain (BrainSeq and UKBEC)^78–80^, blood (eQTLGen, BIOSQTL, and FHS_eQTL)^81–83^, and multiple tissues (GTEx)^84^. To gain a deeper understanding of how genetic variants interact with nongenetic risk factors and explain how they functionally affect the AAO, we searched HaploReg version 4.1 to assess whether the SNPs were located in the regulatory elements^85^. We also mined the Phenoscanner version 2 database to evaluate whether the SNPs were associated with DNA methylation^27^.

### Reporting summary

Further information on research design is available in the Nature Research Reporting Summary linked to this article.

### Supplementary information

Supplementary material

Reporting Summary

## Data Availability

The UK Biobank data are available through the UK Biobank Access Management System https://www.ukbiobank.ac.uk/. eQTLs data are available through the following websites: BrainSeq: http://eqtl.brainseq.org/, UKBEC: http://www.braineac.org/, eQTLGen: https://www.eqtlgen.org/, BIOSQTL: http://genenetwork.nl/biosqtlbrowser, FHS_eQTL: ftp://ftp.ncbi.nlm.nih.gov/eqtl/original_submissions/FHS_eQTL/, and GTEx: http://gtexportal.org.

## Code availability

The code used for the association analysis is available in the git repository: https://github.com/huangyuanf/AAOPD.
