# Supplementary material for: Risk factors associated with age at onset of Parkinson’s disease in the UK Biobank
Source: NPJ Parkinsons Dis. 2024 Jan 2;10:3. doi: 10.1038/s41531-023-00623-9 (PMC10762149; doi:10.1038/s41531-023-00623-9)
Supplement: Supplementary file 1 — Supplementary material [file 41531_2023_623_MOESM1_ESM.pdf]

Supplementary figure 1. QQ plot of the results of the genome-wide environment interaction study for AAO of PD

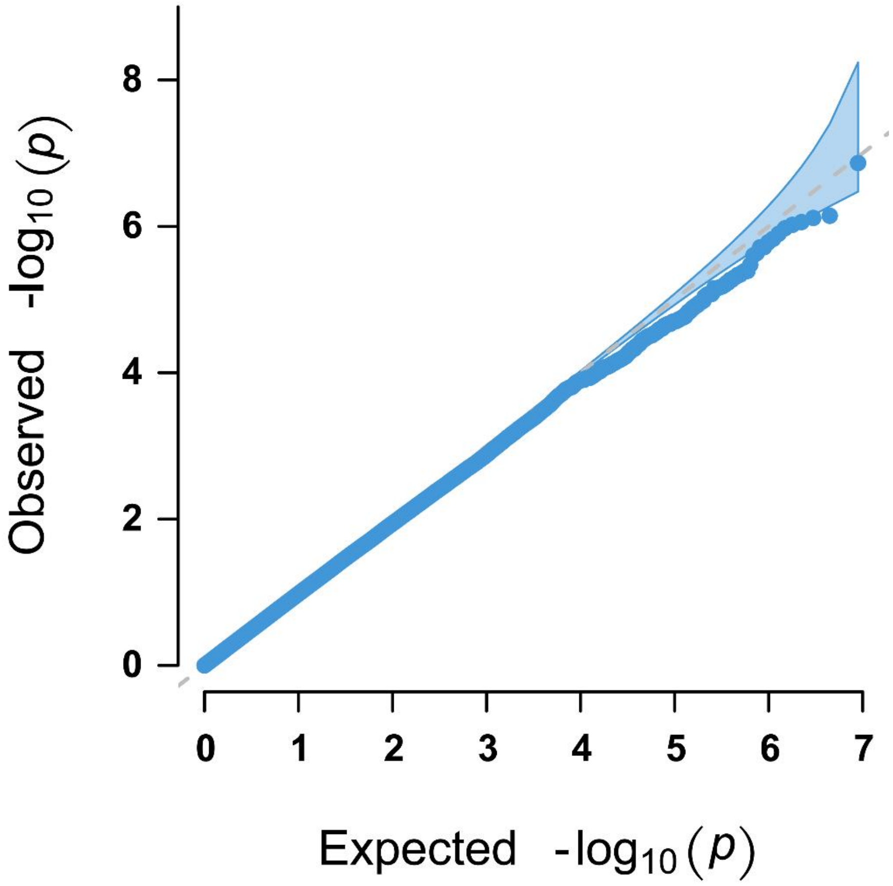

Quantile-quantile (QQ) plot for the tests of interaction between individual SNPs and smoking on AAO of PD. The blue dots denote the distribution of observed –log10(P) values against the expected –log10(P) values from a theoretical chisquare distribution with one df. The straight line represents the theoretical distribution of expected –log10(P) values under the null hypothesis of no association. There was no evidence of any systematic bias: the genomic inflation factor (λ) was equal to 0.972.

Supplementary Table 1. Demographics of study participants

	PD
N	3786
Number of men (%)	2369(62.57%)
Mean age at recruitment (SD)	62.85(±5.35)
Mean AAO (SD)	68.22(±8.90)
Mean Townsend Deprivation index (SD)	-1.4199(±3.0586)
Median age at recruitment (IQR)	64(60~67)
Median AAO (IQR)	70(63~75)
Median Townsend Deprivation index (IQR)	-2.3007(-3.7274~0.4773)

N = number of individuals

AAO = age at onset

IQR = Interquartile Range

SD = Standard Deviation

Supplementary Table 2. Sensitivity analysis of association of non-genetic risk factors with AAO of PD

Variable		Yes	No	P	Adjusted_P
Normal BMI					
	N	842	2195		
	Median AAO (IQR)	71.0(62.0-75.0)	71.0(64.0-75.0)	0.0916	0.6362
Smoking					
	N	1410	1647		
	Median AAO (IQR)	72.0(66.0-76.0)	69.0(62.0-75.0)	**<0.0001***	**<0.0001***
Coffee consumption					
	N	2459	598		
	Median AAO (IQR)	71.0(63.0-75.0)	70.0(63.0-75.0)	0.0982	0.8602
Tea consumption					
	N	2643	410		
	Median AAO (IQR)	71.0(63.0-75.0)	70.5(63.0-75.0)	0.9851	0.2143
Alcohol consumption					
	N	2900	156		
	Median AAO (IQR)	71.0(63.0-75.0)	70.0(63.0-76.0)	0.8546	0.355
Cheese consumption					
	N	2873	96		
	Median AAO (IQR)	71.0(63.0-75.0)	71.0(66.0-75.0)	0.3464	0.1403
Milk consumption					
	N	2965	96		
	Median AAO (IQR)	71.0(63.0-75.0)	71.0(64.0-75.0)	0.8852	0.3592
Moderate or vigorous physical activity					
	N	1691	747		
	Median AAO (IQR)	71.0(64.0-75.5)	69.0(62.0-74.0)	**<0.0001***	**<0.0001***
NSAIDs intake					
	N	994	2027		
	Median AAO (IQR)	72.0(65.0-76.0)	70.0(63.0-75.0)	**<0.0001***	**0.0131***
Excessive daytime sleepiness					
	N	1122	1924		
	Median AAO (IQR)	70.0(61.0-75.0)	71.0(65.0-75.0)	**0.0008***	**<0.0001***
Family history of PD					
	N	266	2797		
	Median AAO (IQR)	68.0(60.0-74.0)	71.0(64.0-75.0)	**0.0002***	**0.0001***

Abbreviations: Median AAO, median age at onset of PD; IQR, interquartile range; N, number of individuals; NA, not applicable; Normal BMI: yes = person with normal BMI, no = person with abnormal BMI; Smoking: yes = person currently or ever smoke; no = person never smoke; Coffee consumption: yes = person drink more than zero cup of coffee each day; no = person

drink zero cup of coffee each day; Tea consumption: yes = person drink more than zero cup of tea each day; no = person drink zero cup of tea each day; Alcohol consumption: yes = person currently or ever drink; no = person never drink; Cheese consumption: yes = person eat cheese; no = person don't eat cheese; Milk consumption: yes = person drink milk; no = person don't drink milk; Moderate or vigorous physical activity: yes = person with metabolic equivalent (MET) minutes per week more than or equal to 600; no = person with metabolic equivalent (MET) minutes per week less than 600; NSAIDs intake: yes = person regularly take in aspirin or ibuprofen; no = person don't take in any aspirin or ibuprofen; P = two-sided exploratory P values from Mann-Whitney U-tests. Adjusted_P = two-sided exploratory P values from generalized linear models adjusted for AAR, gender, and Townsend Deprivation index. Significant associations ($p<0.05$) are in bold with star.

Supplementary Table 3. Loci showing genome-wide interaction (P<=5e-6) with smoking on AAO of PD

CHR	SNP	Closest gene (distance of SNP to gene in kb)	OR	P
1q32	rs4951056	*PLEKHA6* (0)	4.58	4.87×10^{-6}
2q31	rs35262123	*ITGA4* (60kb)	4.93	8.74×10^{-7}
	rs11690881	*ITGA4* (64kb)	4.86	1.26×10^{-6}
5p14	rs116342784	*LOC729862* (20kb)	5.28	1.36×10^{-7}
	rs114720350	*LOC729862* (21kb)	4.95	7.67×10^{-7}
	rs142720753	*LOC729862* (50kb)	4.80	1.64×10^{-6}
	rs72755600	*LOC340107* (60kb)	4.59	4.56×10^{-6}
8q23	rs10093967	*ANGPT1* (0)	-4.97	7.17×10^{-7}
	rs10095329	*ANGPT1* (0)	-4.89	1.06×10^{-6}
	rs1030693	*ANGPT1* (0)	-4.73	2.33×10^{-6}
9q22	rs150424154	*NR4A3* (40kb)	-4.72	2.50×10^{-6}
16p13	rs113991597	*FOPNL* (15kb)	4.82	1.48×10^{-6}
	rs56081915	*FOPNL* (15kb)	4.66	3.33×10^{-6}
	rs7188008	*FOPNL* (17kb)	4.62	4.06×10^{-6}
6q21	rs9934772	*LOC283867* (50kb)	-4.77	1.93×10^{-6}
	rs1423794	*LOC283867* (64kb)	-4.62	4.06×10^{-6}
	rs11644004	*LOC283867* (64kb)	-4.61	4.26×10^{-6}
	rs28802661	*LOC124903780* (1kb)	-4.59	4.60×10^{-6}
17q21	rs540268581	*CA10* (1kb)	4.91	9.54×10^{-7}

Abbreviations: CHR: chromosome; kb: distance to closet gene in kilobases (hg38); OR = Odds ratio; P = generalized linear models adjusted for AAR, gender, Townsend Deprivation index, and PCs 1-4.

Supplementary Table 4. Function annotations of SNPs at suggestive loci interacting with smoking

Locus	SNP	r^2 with lead SNP	Closest gene (distance of SNP to gene in kb)	Cis-eQTL gene	Cis-eQTL Tissue	Cis-eQTL study	Regulatory elements[a] Promoter histones marks	Enhancer histones marks	DNase I hypersensitivity site	Transcription factor binding sites
1q32	**rs4951056**	Lead SNP	*PLEKHA6* (0)	*GOLT1A*	Pancreas; Caudate (basal ganglia); Substantia nigra	GTEx	Yes (skeletal muscle male)	Yes (Brain Substantia Nigra)	Yes (Fetal Brain)	*ARD3A, DBX1, DBX2, HLX1, HMX-2, HOXB13, HOXB9, HOXD8, MEF2, NCX-2, NKX6-1, POU3F4, POU4F3, POU6F1, SOX-2, ZFP105*
				PLEKHA6	Blood; Dorsal Lateral Prefrontal Cortex; Occipital Cortex; Putamen	BIOSQTL; eQTLGen; BrainSeq; UKBEC				
				MDM4	Hippocampus	BrainSeq; UKBEC				
	rs4073136	0.94	*PLEKHA6* (0)	*GOLT1A*	Pancreas; Caudate (basal ganglia); Substantia nigra; Blood; Dorsal Lateral Prefrontal Cortex; Occipital Cortex;	GTEx; BIOSQTL; eQTLGen; BrainSeq; UKBEC	No	Yes (Brain Substantia Nigra)	No	*AHR::ARNT::HIF 1, EGR-1, HIF1, NRF1, PAS-4, SRF, ZFP161-3*

					Putamen				
				MDM4	Hippocampus	BrainSeq; UKBEC				
2q31	**rs35262123**	Lead SNP	*ITGA4* (60kb)	*ITGA4; CERKL;*	Blood; Frontal Cortex; Occipital Cortex; Hippocampus; Esophagus - Mucosa	FHS_eQT; eQTLGen; GTEx; UKBEC	No	Yes (hematopoietic stem cells)	No	*GFI1-1, HDX, NRF1*
				UBE2E3	Blood; Hippocampus; White Matter	FHS_eQT; eQTLGen; UKBEC				
	rs11690881	0.95	*ITGA4* (64kb)	*ITGA4; CERKL;*	Blood; Frontal Cortex; Occipital Cortex; Hippocampus; Esophagus - Mucosa	FHS_eQT; eQTLGen; GTEx; UKBEC	No	Yes (Neuronal Progenitor Cultured Cells)	No	*MYC, SREBP*
				UBE2E3	Blood; Hippocampus; White Matter	FHS_eQT; eQTLGen; UKBEC				
	rs17270903	0.82	*ITGA4* (62kb)	*ITGA4; CERKL;*	Blood; Frontal Cortex; Occipital Cortex; Hippocampus; Esophagus -	FHS_eQT; eQTLGen; GTEx; UKBEC	No	Yes (blood cells)	No	*EVI-1, HDX, OSF2-2, PLZF, STAT*

					Mucosa					
				UBE2E3	Blood; Hippocampus; White Matter	FHS_eQT; eQTLGen; UKBEC				
5p14	**rs116342784**	Lead SNP	*LOC729862* (220kb)	-		-	No	No	No	*DMRT3, FOXP1, HNF4, PLZF, SOX-3, SP100*
8q23	**rs10093967**	Lead SNP	*ANGPT1* (0)	*ANGPT1*	Blood; Lung; Frontal Cortex; Occipital Cortex; Temporal Cortex	eQTLGen; GTEx; UKBEC	No	No	No	*MXI1, MYC*
				OXR1	Cerebellum； Occipital Cortex; Hippocampus	UKBEC				
	rs10095329	0.98	*ANGPT1* (0)	*ANGPT1*	Blood; Lung; Frontal Cortex; Occipital Cortex; Temporal Cortex	eQTLGen; GTEx; UKBEC	No	No	No	*ETS, MYF-4, ZEB1, ZNF143*
				OXR1	Cerebellum； Occipital Cortex; Hippocampus	UKBEC				
9q22	**rs150424154**	Lead SNP	*NR4A3* (40kb)	*INVS*	Blood	eQTLGen	No	Yes (Neuronal	Yes (Trophobla	*CAC, CCNT2, E2F, ETS,*

				STX17	Blood; Esophagus-Mucosa	eQTLGen; GTEx		Progenitor Cultured Cells)	st Cultured Cells)	*INSM1, MAZR, MZF1, SP1, UF1H3BETA, WT1, ZFP281*
	rs183042314	1	*NR4A3* (60kb)	*INVS*	Blood	eQTLGen	Yes (hematopoietic stem cells)	Yes (hematopoietic stem cells)	No	*GCNF*
				STX17	Blood; Esophagus-Mucosa	eQTLGen; GTEx				
16p13	**rs113991597**	Lead SNP	*FOPNL* (15kb)	*FOPNL*	Blood; Muscle - Skeletal	eQTLGen; GTEx	No	No	No	*Brachyury-2, LBP-1,TBX5-2,TBX5-3*
				KIAA0430	Substantia nigra; Putamen	UKBEC				
				MYH11	Substantia nigra; Putamen; Occipital Cortex	UKBEC				
	rs56081915	0.99	*FOPNL* (16kb)	*FOPNL*	Blood; Muscle - Skeletal	eQTLGen; GTEx	No	No	No	*GR, TATA, THAP1, YY1*
				KIAA0430	Substantia nigra; Putamen	UKBEC				
				MYH11	Substantia nigra; Putamen; Occipital Cortex; Frontal Cortex	UKBEC				
	rs7189205	0.99	*FOPNL*	*FOPNL*	Blood	eQTLGen	No	No	No	*E2A-1,E2A-2,E2A*

			(18kb)	*KIAA0430*	Substantia nigra	UKBEC				*-5, MYF-4, NRSF, SEF-1*
				MYH11	Putamen; Occipital Cortex; Frontal Cortex; Substantia nigra	UKBEC				
	rs7188008	0.99	*FOPNL* (18kb)	*FOPNL*	Blood	eQTLGen	No	No	No	*EOMES, SREBP*
				KIAA0430	Substantia nigra; Putamen	UKBEC				
				MYH11	Putamen; Occipital Cortex; Frontal Cortex; Substantia nigra	UKBEC				
16q21	**rs9934772**	Lead SNP	*LOC283867* (50kb)	*CDH11*	Heart - Atrial Appendage; Substantia nigra; Cerebellum; Frontal Cortex; Hippocampus	GTEx; UKBEC	No	Yes (Neuronal Progenitor Cultured Cells)	Yes (Neuronal Progenitor Cultured Cells)	*FOXJ2-2, NRSF*
	rs7189335	1	*LOC283867* (61kb)	*SPINT3*	Blood	GTEx; FHS_eQTL	No	Yes (HUES6 Cells)	No	*MEF2*
				CDH11	Substantia nigra; Cerebellum; Frontal Cortex; Hippocampus	GTEx; FHS_eQTL; UKBEC				

17q21	**rs540268581**	Lead SNP	*CA10* (1kb)	-	-	Yes (muscle of leg)	Yes (skeletal muscle tissue)	No	*MTF1, SRF, ZFP105*

Abbreviations: r^2, linkage disequilibrium measure between a proxy and the lead SNP (in bold) at a locus; kb, kilobase (hg 38).

[a] When SNPs colocalized with regulatory elements, “Yes” is indicated in the corresponding column (data from Haploreg version 4.1).

Supplementary Table 5. DNA Methylation level of SNPs at suggestive loci interacting with smoking

					DNA Methylation			
Locus	SNP	r^2 with lead SNP	Position (kb)	Closest gene (distance of SNP to gene in kb)	CpG site (position, kb)	Tissue	2-sided P[a] (Effect allele: Direction of association)	Study or PMID
1q32	**rs4951056**	Lead SNP	204263	*PLEKHA6* (0)	cg26825650 (204182)	Blood	1.98e-10 (C: NA)	BIOSQTL
						Cord Blood	1.91e-10 (C: positive)	ARIES
					cg00358936 (204165)	Human Immune Cells	4.36e-04 (C: positive)	27863251
	rs4073136	0.94	204265	*PLEKHA6* (0)	cg26825650 (204182)	Cord Blood	1.17e-10 (G: negative)	ARIES
2q31	**rs35262123**	Lead SNP	181396	*ITGA4* (60kb)	cg23529896 (182246)	Blood	3.18e-09 (A: NA)	BIOSQTL
					cg17314228 (182237)	Blood	1.43e-05 (A: NA)	BIOSQTL
	rs11690881	0.95	182257	*ITGA4* (64kb)	cg23529896 (182246)	Blood	3.83e-09 (A: NA)	BIOSQTL
					cg02609279 (182325)	Blood	3.83e-09 (A: NA)	BIOSQTL
					cg17314228 (182237)	Blood	1.51e-05 (A: NA)	BIOSQTL
	rs17270903	0.82	181394	*ITGA4* (62kb)	cg23529896 (182246)	Blood	2.03e-05 (A: NA)	BIOSQTL
					cg17314228 (182237)	Blood	2.61e-05 (A: NA)	BIOSQTL
5p14	**rs116342784**	Lead SNP	28707	*LOC729862*	-	-	-	-

				(220kb)				
8q23	**rs10093967**	Lead SNP	107319	*ANGPT1* (0)	cg15724328 (108314)	Blood	2.51e-13 (C: positive)	ARIES
						Cord Blood	5.76e-12 (C: negative)	ARIES
					cg09396217 (108510)	Human Immune Cells	5.06e-05 (C: positive)	27863251
	rs10095329	0.98	107323	*ANGPT1* (0)	cg15724328 (108314)	Blood<br>Cord Blood	2.51e-13 (C: positive)<br>5.76e-12 (C: negative)	ARIES<br>ARIES
					cg09396217 (108510)	Human Immune Cells	1.09e-06 (C: positive)	27863251
9q22	**rs150424154**	Lead SNP	99783	*NR4A3* (40kb)	-	--	-	-
	rs183042314	1	99762	*NR4A3* (40kb)	-	--	-	-
16p13	**rs113991597**	Lead SNP	15903	*FOPNL* (15kb)	cg00425764 (15949)	Blood	1.15e-11 (A: NA)	BIOSQTL
					cg03737209 (NA)	Human Immune Cells	3.29e-04 (A: negative)	27863251
	rs56081915	0.99	15904	*FOPNL* (16kb)	cg00425764 (15949)	Blood	1.61e-11 (A: NA)	BIOSQTL
	rs7189205	0.99	15906	*FOPNL* (18kb)	cg00425764 (15949)	Blood	4.42e-11 (C: NA)	BIOSQTL
					cg03737209 (NA)	Human Immune Cells	3.29e-04 (C: positive)	27863251
	rs7188008	0.99	15906	*FOPNL* (18kb)	cg00425764 (15949)	Blood	5.66e-11 (A: NA)	BIOSQTL
					cg03737209	Human	3.29e-04 (A: negative)	27863251

					(NA)	Immune Cells		
16q21	**rs9934772**	Lead SNP	65225	*LOC283867* (50kb)	cg08757695 (65256)	Blood	5.41e-126 (C: NA)	BIOSQTL
						Blood	1.31e-24 (C: positive)	ARIES
						Cord Blood	6.17e-13 (C: positive)	ARIES
					cg00769251 (64984)	Human Immune Cells	4.21e-07 (C: negative)	27863251
	rs7189335	1	65223	*LOC283867* (61kb)	cg08757695 (65256)	Blood	3.26e-125 (C: NA)	BIOSQTL
						Blood	1.31e-24 (C: positive)	ARIES
						Cord Blood	6.17e-13 (C: positive)	ARIES
					cg00769251 (64984)	Human Immune Cells	4.21e-07 (C: negative)	27863251
17q21	**rs540268581**	Lead SNP	51628	CA10 (1kb)	-	-	-	-

Abbreviations: r^2, linkage disequilibrium measure between a proxy and the lead SNP (in bold) at a locus; kb, kilobase (hg 38).

[a]P is the P-value for association of SNP with DNA methylation levels at a CpG site (data from the Phenoscanner V2 database).

Direction of association, methylation level of SNP (NA: unclear; negative: lower; positive: higher).
