# Supplementary material for: Risk factors associated with age at onset of Parkinson’s disease in the UK Biobank
Source: NPJ Parkinsons Dis. 2024 Jan 2;10:3. doi: 10.1038/s41531-023-00623-9 (PMC10762149; doi:10.1038/s41531-023-00623-9)
Supplement: Supplementary file 2 — Reporting Summary [file 41531_2023_623_MOESM2_ESM.pdf]

nature portfolio

Corresponding author(s): Bin Li and Jinchen Li

Last updated by author(s): Nov 6, 2023

Reporting Summary

Nature Portfolio wishes to improve the reproducibility of the work that we publish. This form provides structure for consistency and transparency in reporting. For further information on Nature Portfolio policies, see our Editorial Policies and the Editorial Policy Checklist.

Statistics

For all statistical analyses, confirm that the following items are present in the figure legend, table legend, main text, or Methods section.

n/a	Confirmed	
☐	☒	The exact sample size (*n*) for each experimental group/condition, given as a discrete number and unit of measurement
☐	☒	A statement on whether measurements were taken from distinct samples or whether the same sample was measured repeatedly
☐	☒	The statistical test(s) used AND whether they are one- or two-sided<br>*Only common tests should be described solely by name; describe more complex techniques in the Methods section.*
☐	☒	A description of all covariates tested
☐	☒	A description of any assumptions or corrections, such as tests of normality and adjustment for multiple comparisons
☐	☒	A full description of the statistical parameters including central tendency (e.g. means) or other basic estimates (e.g. regression coefficient) AND variation (e.g. standard deviation) or associated estimates of uncertainty (e.g. confidence intervals)
☐	☒	For null hypothesis testing, the test statistic (e.g. *F*, *t*, *r*) with confidence intervals, effect sizes, degrees of freedom and *P* value noted<br>*Give P values as exact values whenever suitable.*
☒	☐	For Bayesian analysis, information on the choice of priors and Markov chain Monte Carlo settings
☒	☐	For hierarchical and complex designs, identification of the appropriate level for tests and full reporting of outcomes
☐	☒	Estimates of effect sizes (e.g. Cohen's *d*, Pearson's *r*), indicating how they were calculated

Our web collection on statistics for biologists contains articles on many of the points above.

Software and code

Policy information about availability of computer code

Data collection	No software was used for data collection.
Data analysis	All statistical analyses and diagrams were performed using R version 4.1.3. To explore the interaction effect of SNPs and smoking on AAO, we used a generalized linear regression model in PLINK2.0 (V.2.00aLM 64-bit Intel)https://www.cog-genomics.org/plink/2.0/.

For manuscripts utilizing custom algorithms or software that are central to the research but not yet described in published literature, software must be made available to editors and reviewers. We strongly encourage code deposition in a community repository (e.g. GitHub). See the Nature Portfolio guidelines for submitting code & software for further information.

Data

Policy information about availability of data

All manuscripts must include a data availability statement. This statement should provide the following information, where applicable:

- Accession codes, unique identifiers, or web links for publicly available datasets
- A description of any restrictions on data availability
- For clinical datasets or third party data, please ensure that the statement adheres to our policy

The UK Biobank data are available through the UK Biobank Access Management System https://www.ukbiobank.ac.uk/. eQTLs data are available through the following websites: BrainSeq: http://eqtl.brainseq.org/, UKBEC: http://www.braineac.org/, eQTLGen: https://www.eqtlgen.org/, BIOSQTL: http://genenetwork.nl/biosqtlbrowser, FHS_eQTL: ftp://ftp.ncbi.nlm.nih.gov/eqtl/original_submissions/FHS_eQTL/, and GTEx: http://gtexportal.org.

Research involving human participants, their data, or biological material

Policy information about studies with human participants or human data. See also policy information about sex, gender (identity/presentation), and sexual orientation and race, ethnicity and racism.

Reporting on sex and gender	Participants used for analysis included both men and women
Reporting on race, ethnicity, or other socially relevant groupings	Individuals of European ancestry
Population characteristics	Individuals of European ancestry
Recruitment	This research has been conducted using the UK Biobank Resource under approved application numbers 103082.
Ethics oversight	UK Biobank has approval from the North West Multi-centre Research Ethics Committee (MREC)

Note that full information on the approval of the study protocol must also be provided in the manuscript.

Field-specific reporting

Please select the one below that is the best fit for your research. If you are not sure, read the appropriate sections before making your selection.

☒ Life sciences ☐ Behavioural & social sciences ☐ Ecological, evolutionary & environmental sciences

For a reference copy of the document with all sections, see nature.com/documents/nr-reporting-summary-flat.pdf

Life sciences study design

All studies must disclose on these points even when the disclosure is negative.

Sample size	We included sample with Parkinson's disease in UK Biobank. PD patients were defined using the International Statistical Classification of Diseases and Related Health Problems 10th Revision (ICD10) code G20 from data field 41202, and self-reported PD cases with code 1262 were identified from data field 20002. After exclusions, a total of 3,156 patients with PD from the UK Biobank were included in this study.
Data exclusions	One individual was randomly selected from a pair of related individuals with first-, second-, or third-degree relatives. Additionally, individuals of non-European ancestry were excluded to reduce bias due to demographic histories.
Replication	NA
Randomization	NA
Blinding	NA. Participants were not allocated to experimental groups during recruitment. The UK Biobank study has collected many hundreds of phenotypes . Thus, there is no designated 'treatment' and 'control' groups, and many types of statistical analyses are possible.

Reporting for specific materials, systems and methods

We require information from authors about some types of materials, experimental systems and methods used in many studies. Here, indicate whether each material, system or method listed is relevant to your study. If you are not sure if a list item applies to your research, read the appropriate section before selecting a response.

Materials & experimental systems

n/a	Involved in the study	
☒	☐	Antibodies
☒	☐	Eukaryotic cell lines
☒	☐	Palaeontology and archaeology
☒	☐	Animals and other organisms
☒	☐	Clinical data
☒	☐	Dual use research of concern
☒	☐	Plants

Methods

n/a	Involved in the study	
☒	☐	ChIP-seq
☒	☐	Flow cytometry
☒	☐	MRI-based neuroimaging

Plants

Seed stocks

Report on the source of all seed stocks or other plant material used. If applicable, state the seed stock centre and catalogue number. If plant specimens were collected from the field, describe the collection location, date and sampling procedures.

Novel plant genotypes

Describe the methods by which all novel plant genotypes were produced. This includes those generated by transgenic approaches, gene editing, chemical/radiation-based mutagenesis and hybridization. For transgenic lines, describe the transformation method, the number of independent lines analyzed and the generation upon which experiments were performed. For gene-edited lines, describe the editor used, the endogenous sequence targeted for editing, the targeting guide RNA sequence (if applicable) and how the editor was applied.

Authentication

Describe any authentication procedures for each seed stock used or novel genotype generated. Describe any experiments used to assess the effect of a mutation and, where applicable, how potential secondary effects (e.g. second site T-DNA insertions, mosiacism, off-target gene editing) were examined.
